# Clarithromycin Plus Intravenous Immunoglobulin Therapy Can Reduce the Relapse Rate of Kawasaki Disease: A Phase 2, Open‐Label, Randomized Control Study

**DOI:** 10.1161/JAHA.116.005370

**Published:** 2017-07-06

**Authors:** Etsuro Nanishi, Hisanori Nishio, Hidetoshi Takada, Kenichiro Yamamura, Mitsuharu Fukazawa, Kenji Furuno, Yumi Mizuno, Kenjiro Saigo, Ryo Kadoya, Noriko Ohbuchi, Yasuhiro Onoe, Hironori Yamashita, Hideki Nakayama, Takuya Hara, Takuro Ohno, Yasuhiko Takahashi, Ken Hatae, Tatsuo Harada, Takayuki Shimose, Junji Kishimoto, Shouichi Ohga, Toshiro Hara

**Affiliations:** ^1^ Department of Pediatrics Graduate School of Medical Sciences Kyushu University Fukuoka Japan; ^2^ Department of Perinatal and Pediatric Medicine Graduate School of Medical Sciences Kyushu University Fukuoka Japan; ^3^ Department of Research and Development of Next Generation Medicine Graduate School of Medical Sciences Kyushu University Fukuoka Japan; ^4^ Center for the Study of Global Infection Kyushu University Hospital Fukuoka Japan; ^5^ Kawasaki Disease Center Fukuoka Children's Hospital Fukuoka Japan; ^6^ Department of Pediatrics Yamaguchi Red Cross Hospital Yamaguchi Japan; ^7^ Department of Pediatrics National Hospital Organization Kokura Medical Center Kitakyushu Japan; ^8^ Department of Pediatrics Fukuoka Higashi Medical Center Koga Japan; ^9^ Department of Pediatrics Oita Prefectural Hospital Oita Japan; ^10^ Department of Pediatrics Japan Community Healthcare Organization (JCHO) Kyushu Hospital Kitakyushu Japan; ^11^ Department of Pediatrics Fukuoka Red Cross Hospital Fukuoka Japan; ^12^ Clinical Research Support Center Kyushu Fukuoka Japan; ^13^ Fukuoka Children's Hospital Fukuoka Japan

**Keywords:** biofilm, clarithromycin, clinical trial, Kawasaki disease, pediatric, relapse, Pediatrics, Clinical Studies

## Abstract

**Background:**

We previously reported that biofilms and innate immunity contribute to the pathogenesis of Kawasaki disease. Therefore, we aimed to assess the efficacy of clarithromycin, an antibiofilm agent, in patients with Kawasaki disease.

**Methods and Results:**

We conducted an open‐label, multicenter, randomized, phase 2 trial at 8 hospitals in Japan. Eligible patients included children aged between 4 months and 5 years who were enrolled between days 4 and 8 of illness. Participants were randomly allocated to receive either intravenous immunoglobulin (IVIG) or IVIG plus clarithromycin. The primary end point was the duration of fever after the initiation of IVIG treatment. Eighty‐one eligible patients were randomized. The duration of the fever did not differ between the 2 groups (mean±SD, 34.3±32.4 and 31.1±31.1 hours in the IVIG plus clarithromycin group and the IVIG group, respectively [*P*=0.66]). The relapse rate of patients in the IVIG plus clarithromycin group was significantly lower than that in the IVIG group (12.5% versus 30.8%, *P*=0.046). No serious adverse events occurred during the study period. In a post hoc analysis, the patients in the IVIG plus clarithromycin group required significantly shorter mean lengths of hospital stays than those in the IVIG group (8.9 days versus 10.3 days, *P*=0.049).

**Conclusions:**

Although IVIG plus clarithromycin therapy failed to shorten the duration of fever, it reduced the relapse rate and shortened the duration of hospitalization in patients with Kawasaki disease.

**Clinical Trial Registration:**

URL: http://www.umin.ac.jp/ctr/index.htm. Unique identifier: UMIN000015437.

**Clinical Perspective**

**What Is New?**

- This is the first clinical trial to evaluate the effect of clarithromycin in patients with Kawasaki disease.
- Although intravenous immunoglobulin plus clarithromycin therapy failed to shorten the duration of fever, it reduced the relapse rate and shortened the duration of hospitalization.

**What Are the Clinical Implications?**

- Intravenous immunoglobulin plus clarithromycin therapy can be effective in reducing the relapse rate in patients of Kawasaki disease.
- Clarithromycin might be effective not only in patients with Kawasaki disease during the acute phase, but also for the prophylaxis of those who are at high risk for developing Kawasaki disease.

## Introduction

Kawasaki disease (KD) is the leading cause of acquired heart disease in childhood among developed countries and is characterized by systemic vasculitis, which predominantly affects the coronary arteries.^1^, ^2^, ^3^ The cause of KD remains unclear, and the incidence is still increasing without any effective prevention method.^4^ Over 20% of patients with KD develop coronary artery aneurisms without proper treatment.^5^ Although the standard therapy for KD, high‐dose intravenous immunoglobulin (IVIG) plus aspirin, has been shown to reduce the incidence of coronary artery abnormalities, the mechanism of the effect has not yet been clarified.^6^, ^7^

The clinical and laboratory features suggest that innate immunity strongly contributes to the pathogenesis of KD.^2^, ^8^ We recently reported that FK565, an innate immune ligand for nucleotide‐binding oligomerization domain–containing protein 1, induced site‐specific inflammation in arteries, including the coronary arteries, in mice via proinflammatory cytokine production and contiguous macrophage accumulation.^9^, ^10^ We also detected some KD‐specific molecules in the sera of patients with KD by liquid chromatography‐mass spectrometry and found that the structure of some of these molecules were similar to those of microbe‐associated molecular patterns (MAMPs) from *Yersinia pseudotuberculosis* and airborne bacteria.^11^ These MAMPs induced proinflammatory cytokine production from human coronary artery endothelial cells.^11^ Furthermore, the proinflammatory cytokine production was markedly enhanced when the cells were cultured under biofilm‐forming conditions.^11^ Taken together, these data suggest that MAMPs that can trigger KD development are derived from biofilms.

Recently, many reports have proven the antibiofilm effects of macrolides, including clarithromycin.^12^, ^13^ In addition, clarithromycin is widely used and considered a safe agent for children. Therefore, we assessed the efficacy of clarithromycin for treating KD. Furthermore, there have been several case reports describing an association between *Mycoplasma pneumoniae* infection and the occurrence or severity of KD.^14^, ^15^, ^16^, ^17^ We also assessed the presence of concomitant *M pneumoniae* infection by polymerase chain reaction (PCR) in patients with KD to determine the relationship between the clinical course of KD and *M pneumoniae*, as clarithromycin is known to be an effective antibiotic agent against this bacterium.

## Methods

### Study Design and Patients

We conducted a 2‐group, open‐label, multicenter, randomized, phase 2 trial at 8 hospitals in Japan between October 2014 and September 2015. Eligible patients were children aged between 4 months and 5 years who had been newly diagnosed with KD according to the definition outlined in the Japanese diagnostic guideline for KD.^18^ Patients with KD were enrolled in this study between days 4 and 8 of illness (day 1 was defined as the first day of a fever).

The major exclusion criteria were: (1) patients without a fever at the time of enrollment; (2) patients with coronary artery abnormalities at the time of enrollment; (3) patients with a history of IVIG treatment within 90 days before enrollment; (4) relapsed or recurrent cases of KD; (5) patients with a history of hypersensitivity reactions to macrolides; (6) patients receiving drugs that interact with clarithromycin, or systemic immunosuppressive agents such as steroids at enrollment; (7) patients with comorbid severe bacterial infection; and (8) patients with a prolonged QT interval (QTc ≥450 ms) or other severe underlying disease.

Written informed consent was obtained from the parents or legal guardians of the patients before enrollment. The study was approved by the institutional review boards at all participating institutions.

### Procedures

Patients were randomly assigned to either the IVIG group or the IVIG plus clarithromycin group in a 1:1 ratio. Patients were allocated through dynamic randomization adjusted by the Kobayashi score^19^ (<5 or ≥5) to ensure a balanced allocation of patients at high risk between the 2 groups. Dynamic randomization was performed using the minimization method, incorporating a random element, via a computer‐generated interactive web‐based response system. The Kobayashi score ranges from 0 to 11, with higher scores predicting the IVIG unresponsiveness in Japanese children.^19^ The parameters of the Kobayashi score consists of age (1 point if ≤12 months), days of illness at diagnosis (2 points if ≤4 days), peripheral blood platelet counts (1 point if ≤30×10^4^ μL), neutrophil percentage (2 points if ≥80%), and serum concentration of sodium (2 points if ≤133 mmol/L), asparate aminotransferase (2 points if ≥100 IU/L), and C‐reactive protein (1 point if ≥100 mg/L). Patients, doctors in charge of the patients, and medical staff were not masked to the assignment.

Patients allocated to the IVIG plus clarithromycin group received 10 mg/kg per day of clarithromycin twice daily for 14 days or longer until 7 days after defervescence. All patients in both groups received immunoglobulin (2 g/kg) administered intravenously over 12 to 20 hours as well as 30 mg/kg per day of aspirin, which was reduced to 5 mg/kg per day after defervescence.

We measured the axillary body temperature with a digital thermometer every 4 hours until defervescence. The use of antipyretics other than regular aspirin was not allowed. We defined a fever as an axillary temperature ≥37.5°C and defervescence as a maximum axillary temperature <37.5°C during 24 consecutive hours. Based on the terminology of the Japanese Society of Kawasaki Disease, the occurrence of a fever caused by KD after the initial defervescence was defined as relapse or recurrence. The occurrence of a fever within 14 days after defervescence was defined as relapse and thereafter as recurrence. Rescue treatments were allowed in cases of IVIG resistance (persistent fever after completion of initial IVIG, relapse, and recurrence) and performed according to the decision of the doctors in charge of the patients. Every relapse/recurrence was assessed and confirmed by a blinded independent central review retrospectively.

We obtained echocardiograms, ECGs, and laboratory data at baseline (study day 0), day 2 (1–3), week 1 (6–8), week 2 (13–15), and week 4 (23–33) after randomization. We measured the internal lumen diameters of the coronary artery by 2‐dimensional echocardiography, and the *z* score was calculated.^20^, ^21^ The coronary artery was defined to be abnormal when the coronary artery findings of patients with KD met any of the following criteria: *z* score ≥2.5,^22^ internal diameter ≥3.0 mm in children younger than 5 years, diameter ≥1.5 times greater than that of any adjacent segment, and clearly irregular luminal contour.^23^ Adverse events were collected on a case report form. Each investigator graded the severity of any adverse events according to the Common Terminology Criteria for Adverse Events version 4.0.

Nasopharyngeal swab specimens were collected immediately after enrollment and stored at −20°C. Viral nucleic acid extraction was performed from each sample using GeneAll Ribospin vRD (GeneAll Biotechnology Co), and *M pneumoniae*,* Streptococcus pneumoniae*,* Haemophilus influenzae*,* Chlamydia pneumoniae*,* Legionella pneumophila*, and *Bordetella pertussis* DNA were amplified by multiplex PCR using a Seeplex PneumoBacter ACE Detection kit (Seegene).

The primary end point was the duration of fever after the initiation of IVIG treatment. The secondary end points were: (1) the incidences of relapse and recurrence; (2) additional rescue treatment; (3) coronary artery abnormality; (4) coronary artery *z* score; (5) laboratory data (white blood cell count, hematocrit, and serum concentrations of aspartate aminotransferase, alanine aminotransferase, sodium, and C‐reactive protein) at weeks 1 and 4; and (6) the frequency of adverse events. In a post hoc analysis, we analyzed the duration of hospitalization in both groups and compared the baseline characteristics and clinical outcomes between the patients who did and did not experience relapse/recurrence.

### Statistical Analysis

It was difficult to estimate the number of patients statistically needed to be enrolled in this study to achieve the primary end point, as we had no large‐scale reference data concerning fevers in patients with KD, which were measured at an extremely short interval of 4 hours. Therefore, we estimated the number of patients needed to achieve statistically significant results based on the incidence of coronary artery abnormalities. To have 80% power to detect a reduction in the incidence of coronary artery abnormalities from 20% to 5%, with a 1‐sided α of 20%, the study required 90 participants, including a 10% attrition rate.

Student *t* test was used for analysis of continuous variables, and logistic regression and likelihood ratio test was used for analysis of categorical variables, unless otherwise specified. In addition, as a sensitivity analysis for the primary end point, we calculated a 2‐sided *P* value with Monte Carlo permutation test (100 000 permutations). We also performed the re‐randomization test with ANCOVA for inference under minimization method. We fixed all data except for the treatment labels, and regenerated the randomization sequence using the minimization algorithm for 10 000 times. A 1‐sided *P* value <0.2 was considered statistically significant for the primary analysis, and a 2‐sided *P* value <0.05 was considered statistically significant for the secondary analysis. The safety data were summarized by treatment groups. All statistical analyses were performed using SAS version 9.3 software (SAS Institute Inc). This trial was registered to the UMIN Clinical Trials Registry (UMIN000015437). The randomization, data management, and data analyses were performed by the Clinical Research Support Center Kyushu (Fukuoka, Japan).

## Results

### Participant Characteristics

During the study period, 257 patients were assessed for trial eligibility. Among them, 91 were ineligible: 47 did not meet inclusion criteria and 44 met exclusion criteria. Eighty‐one patients from the 166 eligible patients provided informed consent and underwent randomization. We discontinued enrollment before the number of enrolled patients reached 90 because of the slow patient accrual during the research funding period. Consequently, 40 patients were assigned to the IVIG plus clarithromycin group and 41 to the IVIG group (Figure). Two patients who were allocated in the IVIG group were excluded from the analysis because they were found to be ineligible after randomization (1 had no fever at the beginning of IVIG treatment, and 1 had been misdiagnosed with KD). Two patients in the IVIG plus clarithromycin group were transferred to another hospital and excluded from the primary analysis. The baseline demographics and characteristics were similar between the 2 groups (Table 1). The median age at the time of enrollment was 2.5 years. Participants reported a median fever duration of 4.0 days before entering the study. A total of 56 patients (70.9%) had received antibiotics before entering the study. One patient allocated to the IVIG plus clarithromycin group had received clarithromycin, and the others had received antibiotics other than macrolides.

**Figure 1:**
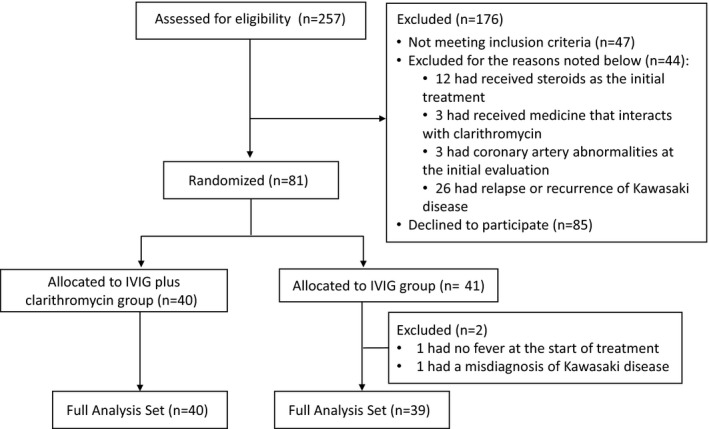
Flow diagram of the study patients. All of the allocated patients received the study drug. IVIG indicates intravenous immunoglobulin.

**Table 1:** Baseline Demographics and Clinical Characteristics

	IVIG+Clarithromycin (n=40)	IVIG (n=39)
Age, y	2.1 (0.6–5.2)	2.6 (0.4–5.6)
Age <1 y	7 (17.5)	7 (17.9)
Men	25 (62.5)	17 (43.6)
Days of illness at enrollment	4.0 (2–8)	5.0 (2–8)
Antibiotics prescribed before enrollment	30 (75.0)	28 (66.7)
Kobayashi risk score^a^	4.0±2.6	3.5±2.7
Kobayashi risk score^a^ ≥5	16 (40.0)	13 (33.3)
Laboratory data		
White blood cell count, 10^9^/L	15.1±5.3	15.0±4.8
Neutrophils, %	70.6±16.7	70.1±13.8
Hematocrit, %	34.1±2.4	34.0±2.5
Aspartate aminotransferase, U/L	136.9±213.9	82.9±132.6
Alanine aminotransferase, U/L	116.6±192.5	76.7±90.5
Sodium, mmol/L	134.9±2.6	134.8±2.7
C‐reactive protein, mg/L	71.5±42.8	79.9±48.4
*z* Scores at enrollment		
Proximal right coronary artery	0.6±1.0	0.7±0.9
Left main coronary artery	0.9±0.9	0.7±0.9
Proximal left anterior descending artery	0.5±1.0	0.4±0.8

Data are median (range), mean±SD, or number (percentage). IVIG indicates intravenous immunoglobulin.

a Kobayashi risk scores range from 0 to 11, with higher scores indicating more severe disease.

### Outcomes

The duration of the fever after the initiation of IVIG treatment, the primary end point, did not differ markedly between the 2 groups (34.3±32.4 in the IVIG plus clarithromycin group and 31.1±31.1 hours in the IVIG group, difference 3.2 hours; 95% CI, −11.7 to 17.7 [*P*=0.66]) (Table 2). The result of Monte Carlo permutation test and re‐randomization test with ANCOVA did not differ from the primary analysis (*P*=0.67 and 0.71, respectively). In contrast, the relapse rate among patients in the IVIG plus clarithromycin group was significantly lower than that in the IVIG group (12.5% versus 30.8%; odds ratio [OR], 0.32; [95% CI, 0.09–0.98]; *P*=0.046) (Table 2). No patients experienced recurrence in either group during the study period. There was no significant difference between the 2 groups in percentage of patients who required additional rescue treatment for KD (Table 2), in the incidence of coronary artery abnormalities, or in the *z* scores at the proximal right coronary artery, left main coronary artery, and proximal left anterior descending coronary artery at week 4 (Table 2). There was no significant difference or any significant effect size for the mean differences in the laboratory data at weeks 1 and 4 after randomization between the 2 groups (Table 3).

**Table 2:** Duration of Fever, Incidence of Treatment Resistance and Additional Treatment, and Coronary Artery Outcomes

	IVIG Plus Clarithromycin		IVIG		Difference or Odds Ratio (95% CI)	*P* Value
	Value	No.	Value	No.		
Primary analysis						
Duration of fever after treatment initiation, h	34.3±32.4	38	31.1±31.1	39	3.2 (−11.2 to 17.7)	0.66
Secondary analysis						
Relapse	5 (12.5)	40	12 (30.8)	39	0.32 (0.09–0.98)	0.046
Recurrence	0	40	0	39	···	···
Additional treatment for Kawasaki disease	11 (27.5)	40	14 (35.9)	39	0.68 (0.26–1.75)	0.42
Coronary artery abnormality at w 4	3 (8.6)	40	2 (5.7)	39	1.55 (0.24–12.33)	0.64
*z* Score of coronary artery at w 4						
Proximal right coronary artery	0.69±1.20	35	0.73±0.98	35	−0.05 (−0.57 to 0.47)	0.86
Left main coronary artery	0.52±0.98	34	0.69±0.90	35	−0.18 (−0.63 to 0.28)	0.44
Proximal left anterior descending artery	0.14±1.10	35	0.23±0.97	35	−0.09 (−0.58 to 0.40)	0.72
Post hoc analysis						
Duration of hospitalization, d	8.9±2.5	38	10.3±3.7	39	−1.4 (−2.9 to 0.0)	0.049

Data are mean±SD or number (percentage). *P* value was calculated using *t* test in analyses of continuous variables and using likelihood ratio test on logistic regression model in analyses of categorical variables. IVIG indicates intravenous immunoglobulin.

**Table 3:** Laboratory Data at Weeks 1 and 4 After Randomization

	IVIG Plus Clarithromycin		IVIG		Cohen's d	*P* Value
	Value	No.	Value	No.		
White blood cell count, 10^9^/L						
Week 1	8.9±2.7	34	10.3±6.2	32	−0.30	0.22
Week 4	8.3±1.9	30	8.6±2.4	30	−0.15	0.57
Neutrophils, %						
Week 1	37.3±13.8	34	43.0±16.8	32	−0.37	0.13
Week 4	34.9±9.0	30	35.2±11.1	30	−0.03	0.90
Hematocrit, %						
Week 1	34.2±3.0	34	33.1±3.0	32	0.39	0.12
Week 4	35.8±2.1	30	35.5±2.2	30	0.12	0.64
Aspartate aminotransferase, U/L						
Week 1	41.1±32.9	34	43.9±36.0	32	−0.08	0.75
Week 4	35.0±5.7	30	37.7±9.2	30	−0.35	0.19
Alanine aminotransferase, U/L						
Week 1	27.4±29.4	34	28.9±30.8	32	−0.05	0.84
Week 4	14.1±4.8	30	18.3±11.6	30	−0.48	0.07
Sodium, mmol/L						
Week 1	137.3±2.0	34	136.8±1.9	32	0.28	0.26
Week 4	139.1±1.9	30	139.1±1.8	30	0.05	0.86
C‐reactive protein, mg/L						
Week 1	6.6±7.6	34	9.3±10.9	32	−0.28	0.26
Week 4	0.7±0.9	30	3.1±10.0	30	−0.34	0.20

Data are mean±SD. Cohen's d was used to describe the standardized mean difference of an effect. *P* values were calculated for between‐group differences by 2‐sided *t* test. IVIG indicates intravenous immunoglobulin.

In a post hoc analysis, the duration of hospitalization of patients in the IVIG plus clarithromycin group was significantly shorter than that in the IVIG group (mean±SD, 8.9±2.5 days versus 10.3±3.7 days; difference −1.4 days [95% CI, −2.9 to 0.0]; *P*=0.049) (Table 2). The high *z* scores for the proximal right coronary artery (OR, 2.03; 95% CI, 1.10–4.07 [*P*=0.02]) and proximal left anterior descending artery (OR, 2.05; 95% CI, 1.09–4.21 [*P*=0.03]) at the time of enrollment were associated with the occurrence of relapse (Table 4), indicating that the dilated coronary artery at the time of enrollment was the risk of relapse. Patients who had a high Kobayashi score of ≥5 points tended to be at risk for relapse, although not statistically significant (OR, 2.75; 95% CI, 0.92–8.46 [*P*=0.07]). The duration of illness before enrollment and the duration of fever after the initiation of IVIG treatment were not associated with the occurrence of relapse (Table 4). Patients who relapsed tended to have longer hospital stays than those who did not relapse (OR, 2.93; 95% CI, 0.96–10.18 [*P*=0.06]). We were unable to detect any association between the rate of relapse and occurrence of coronary artery abnormalities (OR, 2.00; 95% CI, 0.39–8.63 [*P*=0.38]) (Table 4).

**Table 4:** The Effect of Variables at Baseline on the Occurrence of Relapse, and the Effect of Relapse on Clinical Outcomes (Post Hoc Analysis)

	No.	Odds Ratio (95% CI)	*P* Value
Effect of variables on the occurrence of relapse			
Age, y^a^	79	1.28 (0.94–2.08)	0.10
Male sex	79	1.00 (0.34–3.00)	0.98
Days of illness at enrollment^a^	79	0.98 (0.66–1.44)	0.93
Antibiotics prescribed before enrollment	79	3.84 (0.96–25.83)	0.06
Kobayashi risk score ≥5	79	2.75 (0.92–8.46)	0.07
*z* Scores at enrollment			
Proximal right coronary artery^a^	79	2.03 (1.10–4.07)	0.02
Left main coronary artery^a^	79	1.40 (0.75–2.72)	0.29
Proximal left anterior descending artery^a^	79	2.05 (1.09–4.21)	0.03
Duration of fever from treatment initiation to defervescence, h^a^	77	1.00 (0.98–1.02)	0.85
Effect of relapse on clinical outcomes			
Duration of hospital stay ≥9 d (median)	77	2.93 (0.96–10.18)	0.06
Coronary artery abnormality during study period	79	2.00 (0.39–8.63)	0.38

Odds ratios, CIs, and *P* values were obtained from likelihood ratio tests on logistic regression models.

a Odds ratio was interpreted as change in odds per every 1‐unit increment for continuous variable.

### Adverse Events

Adverse events are summarized by group in Table 5. Adverse events were observed in 16 and 12 children in the IVIG plus clarithromycin group and in the IVIG group, respectively. The incidence of grade 3 or 4 adverse events was not significantly different between the IVIG plus clarithromycin group and IVIG group (Table 5). No serious adverse events or deaths occurred during the study period.

**Table 5:** No. of Adverse Events

	IVIG Plus Clarithromycin (n=40)	IVIG (n=38)
All grades	16	12
Grade 3 or 4	1	4
Aspartate aminotransferase increased	1	1
Alanine aminotransferase increased	0	1
Anemia	0	1
Intussusception	0	1

IVIG indicates intravenous immunoglobulin.

### Multiplex PCR for Detection of 6 Pathogens

During the study period, nasopharyngeal swab samples from 68 patients were collected. The results of multiplex PCR are shown in Table 6. All of the samples were tested twice, and the results were the same each time. Among the 68 samples, 57 (83.8%) and 41 (60.3%) were PCR‐positive for *S pneumoniae* and *H Influenzae*, respectively*. M pneumoniae*,* C pneumoniae*,* L pneumophilia*, and *B bertussis* were not detected (Table 6).

**Table 6:** Detection of *M pneumoniae*,* S pneumoniae, H Influenzae, C pneumoniae, L pneumophilia,* and *B bertussis* in Nasopharyngeal Swabs

	*M pneumoniae*	*S pneumoniae*	*H Influenzae*	*C pneumoniae*	*L pneumophilia*	*B bertussis*
Positive	0 (0.0%)	57 (83.8%)	41 (60.3%)	0 (0.0%)	0 (0.0%)	0 (0.0%)
Negative	68 (100.0%)	11 (16.2%)	27 (39.7%)	68 (100.0%)	68 (100.0%)	68 (100.0%)

Data are number (percentage). All of the samples were tested twice and confirmed the concordance. *B bertussis* indicates *Bordetella pertussis; C pneumoniae*,* Chlamydia pneumoniae; H Influenzae, Haemophilus influenza; L pneumophilia, Legionella pneumophila; M pneumoniae*,* Mycoplasma pneumoniae; S pneumoniae, Streptococcus pneumoniae*.

## Discussion

This is the first clinical trial to evaluate the effect of clarithromycin in patients with KD. In this exploratory phase 2 study, the addition of clarithromycin to standard therapy did not affect our primary end point, the duration of the fever. However, clarithromycin plus IVIG therapy significantly reduced the relapse rate and shortened hospital stay compared with IVIG therapy alone.

Clarithromycin, a 14‐membered ring macrolide, is a widely used antibiotic for the treatment of respiratory tract infections.^24^, ^25^ It is well known that clarithromycin and other 14‐ and 15‐membered macrolides have anti‐inflammatory activity that is not mediated through their traditional antimicrobial effect.^12^, ^13^, ^26^, ^27^, ^28^ Biofilm formation and virulence factors are associated with changes in the morphology of bacteria, exopolysaccharide alginate production, and a system of bacterial intercommunication known as quorum sensing. Clarithromycin suppresses the pili assembly, alginate production, and the transcription of several genes that comprise the quorum sensing system, leading to the inhibition of the biofilm formation.^12^, ^13^, ^29^, ^30^, ^31^

Biofilms are medically important and account for over 80% of microbial infections in the body.^32^ The development of toxic shock syndrome, which is an acute systemic shock caused by the exotoxin produced by *Staphylococcus aureus*, is deeply related to biofilm conditions. It is preferentially observed in association with tampon use, wound infection, and burns and other cutaneous lesions.^33^ Furthermore, toxic shock syndrome toxin‐1 production was extremely enhanced when *S. aureus* was cultured as a biofilm rather than cultured with conventional methods, suggesting that *S. aureus* under biofilm condition but not planktonic condition is closely related to the development of toxic shock syndrome.^34^, ^35^ Similarly, we hypothesized that KD may be evoked not by microbes themselves but by bioactive molecules produced by microbes under biofilm‐like conditions.^2^, ^11^ Therefore, we evaluated the clinical effect of clarithromycin as an antibiofilm agent against KD in the current study.

In this study, we found that clarithromycin reduced the relapse rate of KD. Although the precise mechanism of this phenomenon remains to be clarified, we hypothesize that the bioactive KD‐specific MAMPs levels that had decreased after initial IVIG therapy increased again, thereby inducing the relapse of KD. Clarithromycin seemed effective in reducing KD relapse by inhibiting the release of these bioactive molecules via its antibiofilm effect. Therefore, clarithromycin might be effective not only in patients with KD during the acute phase, but also for the prophylaxis of KD in the siblings of patients with KD who are at high risk for developing KD.^36^, ^37^ Recently, Shah et al^38^ reported that the markers of endothelial injury were persistently elevated in patients with KD until a median of 8.3 years after the onset of the disease, even in those without coronary artery abnormalities. Persistent low levels of KD‐specific MAMPs may induce subclinical vasculitis in survivors of KD and cause late‐KD coronary vasculopathy. Clarithromycin might therefore be effective for the long‐term prophylaxis of vasculopathy such as atherosclerosis.

Clarithromycin is widely used to treat certain pulmonary conditions, such as diffuse panbronchiolitis and cystic fibrosis. It improved the pulmonary function in these patients.^39^, ^40^, ^41^ The use of macrolides dramatically improved the prognosis of patients with diffuse panbronchiolitis. Furthermore, clarithromycin lengthened the survival in patients with ventilator‐associated pneumonia and sepsis and shortened the time to the resolution of infection among patients with pyelonephritis, intra‐abdominal infections, and Gram‐negative bacteremia.^42^, ^43^ The mechanisms by which macrolides improve these diseases remain to be clarified. However, mounting evidence suggests that macrolides have immunomodulatory and anti‐inflammatory effects. In addition to its antibiofilm effect, clarithromycin decreases the biosynthesis of proinflammatory cytokines from various cell types.^12^, ^13^, ^26^, ^27^, ^44^, ^45^ Although the mechanism of the effect of clarithromycin on KD could not be determined in the current study, the decrease in the relapse rate may have been mediated by the immunomodulatory effect of clarithromycin.

We assessed the presence of 6 pathogens using a multiplex PCR method to determine the relationship between these pathogens and KD. Several case reports have indicated an association between *M pneumoniae* infection and KD.^14^, ^15^, ^16^, ^17^ In contrast to our expectations, no patients showed positive results for *M pneumoniae* in the present study. Recently, Tang et al^46^ reported that 13.8% (62/450) of patients with KD showed positive results for *M pneumoniae*, which was proven by their increased serum anti–*M pneumoniae* IgM antibody levels and PCR findings. However, those authors found no relationship between the incidence of coronary artery abnormalities and *M pneumoniae*. Furthermore, despite the obvious seasonal peaks in the prevalence of *M pneumoniae* infection in the non‐KD groups, there were no seasonal changes in the number of patients with KD who showed positive findings for *M pneumoniae*.^46^ Our results support their finding that there is no close relationship between *M pneumoniae* and the development of KD.

### Study Limitations

Several limitations associated with the present study warrant mention. First, the relapse rate of our study was relatively high (21.5%). It has been reported that the relapse rate ranges from 3.5% to 22.5%, although the criteria of “relapse” have not been well described.^23^, ^47^, ^48^, ^49^ An epidemiological study indicated changes in the incidence of IVIG‐resistant KD.^50^ The disease severity, which can lead to a high incidence of relapse, might have been higher in our study than in previous studies. Second, we estimated the number of patients needed for this study to achieve statistically significant results based on the incidence of coronary artery abnormalities, but not the primary end point, as we had no large‐scale reference data concerning fevers in patients with KD, which were measured at a relatively short interval of 4 hours. Furthermore, we used a high level of α (20%, 1‐sided) for the sample size calculation and statistical analysis for the primary end point. Pilot or exploratory phase 2 studies designed to evaluate potential efficacy could be conducted with a high level of α up to 25%, which allows a smaller sample size and shorter study period.^51^, ^52^ Although we consider that the high level of α in the current study was permissive, a large‐scale, confirmatory phase 3 study should be designed in more standardized manner. Third, we used minimization, a dynamic allocation method that can balance known prognostic factors between treatment groups. The minimization method is gaining popularity, however, considerable controversy exists over its proper application because it might not be taken as a truly random method.^53^, ^54^ Therefore, we confirmed the robustness of the primary analysis by the sensitivity analysis. Finally, this study used an open‐label design, and none of the patients or researchers were blinded. Furthermore, although the reduction of the relapse rate and duration of hospital stay was statistically significant, the upper limit of the 95% CI of the OR for relapse rate was 0.98 and the upper limit of the 95% CI of the difference for duration of hospitalization was 0.0 day. We could not exclude the possibility of the modest effect of clarithromycin in this small scaled trial. The statistically significant results in this study were obtained by secondary and post hoc analyses, which were unadjusted for multiple testing. We should consider the reproducibility of the results because we cannot ignore the effect of multiple testing. To clarify the precise effect of clarithromycin in patients with KD, a phase 3, double‐blinded, confirmatory study is underway (UMIN000024311).

## Conclusions

This was the first clinical trial to evaluate the effect of clarithromycin in patients with KD, showing that the addition of clarithromycin to standard IVIG therapy did not affect the duration of fever. However, clarithromycin plus IVIG therapy significantly reduced the relapse rate and shortened the hospital stay.

## Sources of Funding

This trial was supported by the Practical Research Project for Allergic Diseases and Immunology (Research on Allergic Diseases and Immunology) from Japan Agency for Medical Research and Development, AMED, and JSPS KAKENHI grant No. JP16K10069.

## Disclosures

None.
